# Psychometric evaluation of the Perceived Stress Scale (PSS-10) in Ecuadorian adults: evidence from a bifactor model and measurement invariance

**DOI:** 10.3389/fpsyg.2026.1936453

**Published:** 2026-08-28

**Authors:** Nairoby Jackeline Pineda-Cabrera, Víctor Manuel López-Guerra, Karina Ocampo-Vásquez

**Affiliations:** Faculty of Behavioral Sciences, Universidad Técnica Particular de Loja, Loja, Ecuador

**Keywords:** bifactor model, Ecuadorian adults, perceived stress, Perceived Stress Scale (PSS-10), psychometric properties

## Abstract

**Background:**

Perceived stress is a key transdiagnostic construct associated with multiple indicators of psychological maladjustment. Although the Perceived Stress Scale (PSS-10) has previously been validated in Ecuador, evidence has been limited to university students. This study examined the psychometric properties of the PSS-10 in a large community sample of Ecuadorian adults.

**Methods:**

A cross-sectional instrumental study was conducted with 16,074 Ecuadorian adults (mean age = 30.91 years, SD = 10.05; 46.8% women). The sample was randomly divided into exploratory (*n* = 8,019) and confirmatory (*n* = 8,055) subsamples. Factorial structure, bifactor reliability, measurement invariance across sex, and validity based on relationships with loneliness, psychological inflexibility, depressive symptoms, and impulsivity were evaluated.

**Results:**

Exploratory analyses supported a two-factor structure (Negative Stress and Coping), whereas confirmatory analyses indicated that a bifactor model, comprising one general perceived stress factor and two specific factors, provided the best fit among the competing models (CFI = 0.990, TLI = 0.982, SRMR = 0.043), although RMSEA remained elevated (0.098; 90% CI [0.096, 0.101]). Bifactor indices supported the predominance of a general factor (ECV = 0.561, ω*H* = 0.645, *H* = 0.940), while the Coping factor retained meaningful reliable variance beyond the general factor. The total score showed excellent reliability (ω = 0.945) and moderate-to-strong positive associations with psychological inflexibility (*r* = 0.711), depressive symptoms (*r* = 0.698), loneliness (*r* = 0.582), and impulsivity (*r* = 0.579). Measurement invariance analyses provided limited evidence of equivalence across sex, and women reported higher observed stress scores than men (*F* = 1,438.81, *p* < 0.001, η^2^ = 0.152).

**Conclusions:**

The Ecuadorian PSS-10 provides reliable and valid scores for assessing perceived stress in adults. The bifactor model supported the use of the total score as an overall indicator of perceived stress, while the Coping factor may provide complementary information. These findings should be interpreted considering the overall psychometric evidence and the limited support for measurement equivalence across sex.

## 1 Introduction

Stress is a complex biopsychosocial construct that arises from the interaction between environmental demands and individuals' perceptions of their ability to cope with them (Lazarus and Folkman, 1984; Juárez-García et al., 2023; Yilmaz Kogar and Kogar, 2024). From this perspective, Cohen et al. (1983) defined perceived stress as the degree to which individuals appraise situations in their lives as unpredictable, uncontrollable, and overwhelming. Accordingly, stress is not determined solely by the objective occurrence of adverse events but also by individuals' cognitive appraisals of environmental demands and their perceived capacity to manage them (Lazarus and Folkman, 1984).

Given its substantial impact on both physical and mental health, stress has become a central construct in psychological and health sciences research (Juárez-García et al., 2023; Santos-Vitti et al., 2024). A growing body of evidence indicates that elevated levels of stress are associated with cardiovascular disease, cognitive impairment, increased mortality risk, and a range of mental health problems, including anxiety, depression, and insomnia (Cohen et al., 2019; Epel and Prather, 2018; Reis et al., 2019; Turner et al., 2020). Furthermore, the World Health Organization (World Health Organization, 2026) recognizes that stress can manifest through physical, emotional, and behavioral symptoms, significantly affecting daily functioning and quality of life.

Stress can be assessed using physiological indicators, behavioral observations, and self-report measures (Crosswell and Lockwood, 2020). Among the latter, perceived stress scales are particularly valuable because they capture individuals' subjective appraisals of environmental demands and their available coping resources (Cohen et al., 1995). One of the most widely used instruments for this purpose is the Perceived Stress Scale (PSS), developed by Cohen et al. (1983), which has become a standard measure of perceived stress worldwide due to its brevity, ease of administration, and sound psychometric properties. Its widespread use and scientific relevance have led to its inclusion in the NIH Toolbox for the assessment of neurological and behavioral functioning (Harris et al., 2023). Compared with longer or more specific instruments used to assess stress such as occupational stress questionnaires focused on workplace demands (e.g., the Job Content Questionnaire) or physiological indicators requiring biological sampling (e.g., cortisol assays), the PSS-10 offers a considerably shorter administration time, does not require occupational status or clinical setting for its application, and captures a global, transdiagnostic appraisal of stress rather than a domain or context specific one. This combination of brevity, ease of self-administration, and applicability across age groups, occupational statuses, and clinical and non-clinical populations has made it one of the most extensively used self-report measures of perceived stress internationally.

The original version of the scale (PSS-14) demonstrated satisfactory levels of internal consistency, temporal stability, and criterion validity (Cohen et al., 1983). Subsequently, Cohen's (1988) concluded that the abbreviated 10-item version (PSS-10) exhibited a more robust factorial structure and superior psychometric performance. Since then, the PSS-10 has been translated, adapted, and validated across a wide range of countries and languages, including Arabic (Chaaya et al., 2010), Chinese (Huang et al., 2020), French (Lesage et al., 2012), German (Bastianon et al., 2020), Japanese (Mimura and Griffiths, 2004), Portuguese (Trigo et al., 2010), and Spanish (Remor, 2006). Across these cultural contexts, the instrument has consistently demonstrated satisfactory reliability and validity.

In addition to its widespread international use, the PSS-10 has been extensively validated across Latin America, demonstrating robust psychometric performance in diverse Spanish-speaking populations. Recent studies conducted in Chile (Jorquera-Gutiérrez and Guerra-Díaz, 2023), Peru (Dominguez-Lara et al., 2022) and Mexico (Juárez-García et al., 2023) have consistently supported its factorial validity and reliability. Collectively, these findings reinforce the PSS-10 as a culturally appropriate and psychometrically sound instrument for assessing perceived stress across Latin American populations.

In Ecuador, Ruisoto et al. (2020) examined the psychometric properties of the PSS-10 and reported satisfactory reliability coefficients (α = 0.85, ω = 0.87). They also found positive associations between perceived stress and loneliness, psychological inflexibility, alcohol consumption, anxiety, and depressive symptoms, as well as negative associations with resilience. These findings support the usefulness of the instrument and provide evidence of validity based on its relationships with theoretically relevant constructs.

Despite its widespread use, considerable debate remains regarding the factorial structure of the PSS-10. Although the scale was originally conceptualized as a unidimensional measure of perceived stress (Cohen's, 1988), numerous studies have reported that a correlated two-factor model comprising Negative Stress and Perceived Coping provides a better fit than a unidimensional solution (Chen et al., 2024; Dominguez-Lara et al., 2022; Jorquera-Gutiérrez and Guerra-Díaz, 2023; Makhubela, 2022; Hakim et al., 2024; Sharif-Nia et al., 2024; Taylor, 2015; Zhang and Wang, 2024). More recently, several authors have argued that a bifactor structure more accurately captures the dimensionality of the scale by allowing a dominant general perceived stress factor to coexist with specific factors primarily associated with positively and negatively worded items (Denovan et al., 2017; Guimarães Freitas et al., 2025; Juárez-García et al., 2023; Reis et al., 2019; Wu and Amtmann, 2013).

Several studies have also demonstrated measurement invariance of the PSS-10 across sex, supporting the comparability of scores between men and women (Chen et al., 2024; Denovan et al., 2017; Jorquera-Gutiérrez and Guerra-Díaz, 2023; Juárez-García et al., 2023; Makhubela, 2022; Reis et al., 2019; Santos-Vitti et al., 2024). However, this psychometric property has received limited attention in Ecuadorian populations. In addition, accumulating evidence suggests that perceived stress may function as a transdiagnostic construct, given its consistent associations with a broad range of indicators of psychological adjustment and maladjustment (Ruisoto et al., 2022; Vaca-Gallegos et al., 2025). Previous research has reported significant relationships between perceived stress and general health and self-esteem (Chen et al., 2024), positive and negative affect (Denovan et al., 2017), psychological negative stress (Guimarães Freitas et al., 2025), resilience (Chen et al., 2024; Guimarães Freitas et al., 2025) depressive symptoms, insomnia severity (Reis et al., 2019), and anxiety (Reis et al., 2019; Remor, 2006). Taken together, these findings highlight the relevance of perceived stress within contemporary transdiagnostic frameworks and underscore the need for further evidence of validity based on its relationships with mental health–related constructs.

Although the PSS-10 has previously been evaluated in Ecuador (Ruisoto et al., 2020), its validation was conducted exclusively in a sample of university students, a population with relatively homogeneous sociodemographic characteristics that limits the generalizability of the findings to the broader Ecuadorian adult population. Furthermore, important psychometric issues remain unresolved, including the adequacy of a bifactor structure, the extent to which a general perceived stress factor accounts for the common variance among items, measurement invariance across sex, and validity evidence based on relationships with transdiagnostic variables in large and heterogeneous community samples. Given the central role of perceived stress in psychological functioning and its well-established associations with multiple indicators of mental health, robust psychometric evidence from the general population is essential to support the use of the PSS-10 in research, clinical practice, and epidemiological studies. Based on previous evidence, it was hypothesized that the bifactor model would demonstrate a superior fit compared with unidimensional and correlated two-factor models, that the scale would exhibit measurement invariance across sex, and that perceived stress scores would be positively associated with loneliness, psychological inflexibility, depressive symptoms, and impulsivity. Accordingly, the present study aimed to examine the psychometric properties of the 10-item Perceived Stress Scale (PSS-10) in a large sample of Ecuadorian adults. Specifically, we evaluated its factorial structure through exploratory and confirmatory factor analyses, its reliability, the adequacy of a bifactor model, measurement invariance across sex, and validity evidence based on its relationships with loneliness, psychological inflexibility, depressive symptoms, and impulsivity.

## 2 Materials and methods

### 2.1 Study design

This study employed an instrumental psychometric design to evaluate the psychometric properties of a psychological assessment instrument (Montero and León, 2002).

### 2.2 Participants

This study employed a cross-sectional design using a non-probabilistic community sample of Ecuadorian adults. Initially, 20,036 participants were recruited through an online survey distributed nationwide across Ecuador. Recruitment was carried out through two complementary strategies. First, study advertisements were disseminated through the websites and institutional communication channels of the Ministry of Public Health of Ecuador, the Secretariat of Higher Education, Science, Technology and Innovation (SENESCYT-SGES-SIES-2021-1156-O), and 48 public and private higher education institutions throughout the country. Second, additional participants were invited via email after attending a series of webinars organized on October 10, 2021, in commemoration of World Mental Health Day.

Data collection was conducted over a 3-month period, from October 2021 to January 2022. Participation was voluntary, anonymous, and no financial compensation was provided. Participants who did not complete the survey were excluded from the analyses, resulting in a final sample of 16,074 adults. No additional participants or items were removed after data screening procedures.

The final sample consisted of 16,074 Ecuadorian adults, including 53.2% men and 46.8% women, with ages ranging from 18 to 72 years (*M* = 30.91, SD = 10.05). Participants were recruited from all four geographical regions of Ecuador, including the Andean region (*n* = 9,791), the Coastal region (*n* = 4,854), the Amazon region (*n* = 1,398), and the Galápagos Islands (*n* = 31), providing broad geographical coverage across the country. The sample also represented diverse marital and employment conditions. Detailed sociodemographic characteristics of the sample are presented in Table 1.

**Table 1:** Sociodemographic Characteristics of the Participants (*N* = 16,074).

Variable	Category	*n*	%
Sex	Men	8,546	53.2
	Women	7,528	46.8
Age	Mean (SD)	30.91 (10.05)	—
	Range	18–72 years	—
Geographical region	Andean region	9,791	60.9
	Coastal region	4,854	30.2
	Amazon region	1,398	8.7
	Galápagos Islands	31	0.2
Marital status	Single	8,728	54.3
	Married/cohabiting	6,446	40.1
	Separated/divorced/widowed	900	5.6
Employment status	Full-time employment	8,999	56.0
	Unemployed	4,453	27.7
	Part-time employment	2,622	16.3

*n*, number of participants; %, percentage of participants within each category.

### 2.3 Measures

#### 2.3.1 Sociodemographic questionnaire

Participants completed a brief sociodemographic questionnaire designed to collect information related to age, sex, marital status, geographical region of residence, and employment status.

#### 2.3.2 Perceived Stress Scale (PSS-10)

Perceived stress was assessed using the 10-item version of the Perceived Stress Scale originally developed by Cohen et al. (1983) and previously adapted for the Ecuadorian population by Ruisoto et al. (2020). The instrument evaluates the extent to which individuals perceive situations in their lives as stressful, unpredictable, or uncontrollable during the last month. Items are rated on a five-point Likert-type scale ranging from 0 (“never”) to 4 (“very often”), with higher scores reflecting greater perceived stress.

Previous research has shown that the PSS-10 has a bifactor structure, comprising a dominant general perceived stress factor and two specific dimensions reflecting Negative Stress and Coping, which explains 56.99% of the total variance and demonstrates satisfactory convergent validity with multiple health indicators (Ruisoto et al., 2020) Consistent with these findings, the same structure was supported in the present study. An example item is: “*In the last month, how often have you felt nervous and stressed?.”*

#### 2.3.3 UCLA loneliness scale–short version

Loneliness was assessed using the short version of the UCLA Loneliness Scale developed by Russell (1996) and subsequently abbreviated by Hughes et al. (2004). This instrument assesses subjective feelings of loneliness and perceived deficiencies in social relationships. Participants respond using a three-point Likert-type scale ranging from 1 (“hardly ever”) to 3 (“often”), with higher scores indicating greater perceived loneliness. Previous research has shown that the three-item version demonstrates adequate internal consistency (Cronbach's α = 0.72), strong agreement with the original 20-item UCLA Loneliness Scale (*r* = 0.82), and satisfactory convergent and discriminant validity, supporting its use as a brief measure of loneliness in large-scale surveys (Hughes et al., 2004). An example item is: “*How often do you feel that you lack companionship?.”* In the present study, the instrument demonstrated satisfactory internal consistency, with a Cronbach's alpha coefficient of α = 0.85.

#### 2.3.4 Acceptance and action questionnaire-II (AAQ-II)

Psychological inflexibility was measured using the Acceptance and Action Questionnaire-II developed by Bond et al. (2011) and adapted for the Ecuadorian population by Paladines-Costa et al. (2021). The AAQ-II is a seven-item self-report instrument designed to assess experiential avoidance and psychological inflexibility. Items are rated on a seven-point Likert-type scale ranging from 1 (“never true”) to 7 (“always true”), with higher scores indicating greater psychological inflexibility. Previous research in Ecuadorian university students demonstrated that the AAQ-II has a unidimensional factor structure, explaining 66.87%−70.03% of the total variance, with excellent internal consistency (Cronbach's α = 0.92; McDonald's ω = 0.93), measurement invariance across sex, and satisfactory convergent and divergent validity with multiple psychological health indicators (Paladines-Costa et al., 2021). An example item is: “*I worry about not being able to control my worries and feelings.”* In the present study, the instrument demonstrated excellent internal consistency, with a Cronbach's alpha coefficient of α = 0.96.

#### 2.3.5 Patient health questionnaire-9 (PHQ-9)

Depressive symptomatology was assessed using the Patient Health Questionnaire-9 (PHQ-9) developed by Kroenke et al. (2001), using the Ecuadorian adaptation reported by López-Guerra et al. (2022) The PHQ-9 consists of nine items that assess the frequency of depressive symptoms associated with major depressive disorder during the previous 2 weeks. Responses are recorded on a four-point Likert scale ranging from 0 (“not at all”) to 3 (“nearly every day”). Total scores range from 0 to 27, with higher scores indicating greater depressive symptom severity. Previous evidence in Ecuadorian university students supports its psychometric adequacy, showing excellent internal consistency (ω = 0.90), a hierarchical structure comprising a general factor and three specific latent factors (somatic, cognitive/affective, and concentration/motor), as well as satisfactory convergent and divergent validity with multiple health indicators (López-Guerra et al., 2022). An example item is: “*Trouble falling or staying asleep, or sleeping too much.”* In the current study, the scale demonstrated excellent internal consistency, with a Cronbach's alpha coefficient of α = 0.94.

#### 2.3.6 Barratt impulsiveness scale-11 (BIS-11)

Impulsivity was assessed using the BIS-11, originally developed by Patton et al. (1995) and adapted into Spanish by Oquendo et al. (2001). The BIS-11 conceptualizes impulsivity as a multidimensional construct characterized by rapid, unplanned reactions to internal or external stimuli without adequate consideration of their consequences. The instrument consists of 30 self-report items assessing three dimensions of impulsivity: attentional (cognitive), motor, and non-planning impulsivity. Items are rated on a four-point Likert-type scale ranging from 1 (“rarely or never”) to 4 (“almost always or always”), with higher scores indicating greater impulsivity. The Spanish adaptation followed a rigorous process of translation, back-translation, and evaluation of linguistic, conceptual, and scale equivalence, providing evidence that the instrument is suitable for use in Spanish-speaking populations (Oquendo et al., 2001). An example item is: “*I act on the spur of the moment.”* In the present study, the instrument exhibited strong internal consistency, with a Cronbach's alpha coefficient of α = 0.89.

### 2.4 Design and procedure

A cross-sectional study design was employed. The study protocol was approved by the Ethics Committee for Research in Human Beings (Comité de Ética de Investigación en Seres Humanos, CEISH) of the Universidad San Francisco de Quito in August 2021 (CEISH: 2021-072E), with support from the Ecuadorian Ministry of Public Health. All procedures were conducted in accordance with the ethical principles established in the Declaration of Helsinki (World Medical Association, 2013).

Data collection was carried out through a web-based survey platform specifically designed for the study. The selected instruments were chosen based on their brevity, psychometric robustness, and suitability for research purposes. Online administration facilitated automatic data recording and score computation, reduced the likelihood of missing data, optimized data collection procedures, and ensured participant anonymity and confidentiality.

Before accessing the questionnaires, participants were presented with an electronic informed consent form detailing the objectives of the study, the voluntary nature of participation, confidentiality guarantees, and ethical considerations. Participants were required to provide informed consent electronically before proceeding to the survey.

The assessment protocol followed the same administration sequence for all participants and included the following instruments: an *ad hoc* sociodemographic questionnaire, the Barratt Impulsiveness Scale-11 (BIS-11), the Perceived Stress Scale (PSS-10), the UCLA Loneliness Scale–Short Version (UCLA-3), the Acceptance and Action Questionnaire-II (AAQ-II) and the Patient Health Questionnaire-9 (PHQ-9).

### 2.5 Data analysis

All exploratory and confirmatory factor analyses were conducted using JASP version 0.95.2.0. Because JASP does not compute several bifactor-specific statistics, the Explained Common Variance (ECV), Percentage of Uncontaminated Correlations (PUC), hierarchical omega (ω*H*), and construct replicability index (*H*) were calculated using the Bifactor Indices Calculator, a Microsoft Excel-based application specifically developed to estimate bifactor indices from confirmatory factor analysis models (Dueber, 2017).

Following methodological recommendations for psychometric validation studies, the total sample (*N* = 16,074) was randomly divided into two independent subsamples to reduce capitalization on chance and to allow cross-validation of the factorial structure. Specifically, the exploratory factor analysis (EFA) was conducted using a subsample of 8,019 participants, whereas the confirmatory factor analysis (CFA) was performed on an independent subsample of 8,055 participants, following the recommendations proposed by Harrington (2008).

Initially, descriptive analyses were conducted for all items to assess the distributional properties of the data. Measures of central tendency, dispersion, skewness, and kurtosis were examined. According to the criteria proposed by Kim (2013), skewness values below |2| and kurtosis values below |7| were considered indicative of acceptable univariate normality.

Before conducting factor analyses, the adequacy of the correlation matrix was evaluated using the Kaiser–Meyer–Olkin (KMO) index and Bartlett's test of sphericity. The EFA was performed using oblimin rotation because the latent dimensions were theoretically expected to be correlated. Factor retention was based on eigenvalues, explained variance, factor loadings, and theoretical interpretability.

To evaluate the factorial structure of the PSS-10, confirmatory factor analyses (CFA) were conducted by comparing competing theoretical models, including a unidimensional model, a correlated two-factor model, and a bifactor model. Given the ordinal nature of the items, analyses were estimated using robust weighted least squares procedures based on polychoric correlations. Model fit was evaluated using the Comparative Fit Index (CFI), Tucker–Lewis Index (TLI), Root Mean Square Error of Approximation (RMSEA), and Standardized Root Mean Square Residual (SRMR). Following the recommendations of Hu and Bentler (1999), values of CFI and TLI ≥ 0.95, RMSEA ≤ 0.08, and SRMR ≤ 0.08 were considered indicative of acceptable model fit.

Because the retained structure corresponded to a bifactor model, additional bifactor-specific indices were calculated, including Explained Common Variance (ECV), Percentage of Uncontaminated Correlations (PUC), omega coefficient (ω), hierarchical omega coefficient (ω*h*), and the construct replicability index (*H*). Following the recommendations of Rodriguez et al. (2016), these indices were used to evaluate the relative contribution of the general and specific factors and to determine the degree of essential unidimensionality of the scale.

Internal consistency was evaluated using omega-based reliability indices, specifically omega coefficient (ω) and hierarchical omega coefficient (ω*h*), because these indicators provide more appropriate reliability estimates for bifactor structures. According to Rodriguez et al. (2016), ω and ω*h* values ≥ 0.70 and ≥ 0.30, respectively, were considered indicative of satisfactory reliability and substantive contribution of the latent dimensions. Construct replicability was additionally examined using the H coefficient, with values above 0.70 indicating adequate latent construct replicability.

Subsequently, measurement invariance across sex was examined using multigroup confirmatory factor analysis (MG-CFA) estimated with the WLSMV estimator for ordered-categorical indicators. Configural, metric, and scalar models were evaluated sequentially. Invariance decisions followed the recommendations of Cheung and Rensvold (2002) and Chen (2007), considering ΔCFI ≤ .010 and ΔRMSEA ≤ .015 as evidence of measurement invariance across groups. Because robust fit statistics obtained with WLSMV estimation may exhibit non-monotonic patterns across nested models, the interpretation emphasized the overall pattern of fit indices and the predefined change-in-fit criteria rather than relying exclusively on chi-square difference tests.

After evaluating factorial invariance, convergent and discriminant validity were examined through Pearson correlation analyses between perceived stress scores and theoretically related external variables, including loneliness, psychological inflexibility, depressive symptoms, and impulsivity. Correlation magnitudes were interpreted according to the criteria proposed by Cohen's (1988), where values of 0.10–0.29 indicate small associations, 0.30–0.49 moderate associations, and values ≥ 0.50 large associations.

Construct validity through the contrasted-groups method was assessed by examining sex differences in PSS-10 scores using one-way analyses of variance (ANOVA). Effect sizes were estimated using eta squared (η^2^), interpreted according to Cohen (1988) guidelines as small (0.01), medium (0.06), and large (0.14).

Finally, descriptive analyses of the latent dimensions derived from the best-fitting bifactor model were conducted. Measures of central tendency, dispersion, quartiles, skewness, kurtosis, and graphical distribution analyses through boxplots were examined to characterize the distributional properties of the total perceived stress score and its specific dimensions (Negative Stress and Coping).

## 3 Results

### 3.1 Exploratory factor analysis

A preliminary analysis indicated that the items of the Perceived Stress Scale (PSS-10) showed acceptable univariate normality. Skewness values ranged from 0.182 to 0.821, indicating a slight positive skew across items; however, all values remained within acceptable limits (|Skew| < 2). Kurtosis values ranged from −1.174 to −0.341, suggesting a platykurtic distribution, but well below the recommended cutoff (|Kurtosis| < 7), thus supporting the assumption of normality (see Table 2). These criteria are consistent with the recommendations proposed by Arenas et al. (2023), who suggest maximum acceptable values of 2 for skewness and 7 for kurtosis. Overall, the distributional properties of the items support their suitability for factor analysis.

**Table 2:** Descriptive statistics and factor loadings for the Perceived Stress Scale.

Items	Mean	SD	Skew	Kurt	Factor 1	Factor 2	Uniq
1	1.202	1.131	0.619	−0.453	0.804		0.257
2	1.100	1.195	0.821	−0.341	0.875		0.156
3	1.746	1.314	0.182	−1.050	0.858		0.222
4	1.759	1.404	0.305	−1.174		0.779	0.363
5	1.658	1.283	0.443	−0.823		0.875	0.231
6	1.336	1.208	0.522	−0.730	0.793		0.370
7	1.695	1.314	0.427	−0.917		0.889	0.207
8	1.671	1.302	0.441	−0.868		0.895	0.196
9	1.421	1.209	0.483	−0.714	0.868		0.241
10	1.220	1.217	0.728	−0.478	0.936		0.106
Percentage of variance					44.7%	29.8%	
Total variance						74.5%	

SD, Standard Deviation; Skew, Skewness; Kurt, Kurtosis; Uniq, Uniqueness (i.e., the proportion of variance not explained by the common factors).

Prior to conducting the exploratory factor analysis (EFA), the adequacy of the data was assessed. The Kaiser–Meyer–Olkin (KMO) measure indicated excellent sampling adequacy (KMO = 0.895), with all individual item values exceeding 0.80. Bartlett's test of sphericity was statistically significant, χ^2^(45) = 67,883.85, *p* < 0.001, confirming that the correlation matrix was suitable for factor analysis.

The EFA was performed using oblimin rotation, given the theoretical expectation of correlated factors. The results revealed a clear and interpretable two-factor structure. The first factor, comprising negatively worded items (perceived negative stress), exhibited strong factor loadings ranging from 0.793 to 0.936 (items 1, 2, 3, 6, 9, and 10). The second factor, representing positively worded items (perceived coping), also showed high loadings, ranging from 0.779 to 0.895 (items 4, 5, 7, and 8). No substantial cross-loadings were observed, and all items loaded clearly onto their respective factors, supporting a well-defined and theoretically coherent structure.

The eigenvalues and explained variance further supported the two-factor solution. The first factor yielded an eigenvalue of 4.47, accounting for 44.7% of the total variance, while the second factor had an eigenvalue of 2.98, explaining an additional 29.8%. Together, both factors accounted for 74.5% of the total variance, indicating a strong representation of the underlying construct.

Item communalities were satisfactory, as reflected by low uniqueness values ranging from 0.106 to 0.370, suggesting that a substantial proportion of variance in each item was explained by the extracted factors.

Overall, these findings provide robust empirical support for a two-factor structure, consistent with the distinction between perceived negative stress and perceived coping. The strength of the factor loadings, the high proportion of explained variance, and the absence of cross-loadings collectively support the structural validity of the scale in this sample.

### 3.2 Confirmatory factor analysis

To examine the factorial structure of the 10-item Perceived Stress Scale (PSS-10), three competing confirmatory factor models were tested and compared (see Table 3).

**Table 3:** Goodness-of-fit indices for the competing confirmatory factor models of the PSS-10.

Modelo	CMIN/df	CFI	TLI	RMSEA	SRMR
M1	2010.95	0.635	0.531	0.500	0.363
M2	122.20	0.979	0.972	0.123	0.069
M3	77.24	0.990	0.982	0.098	0.043

CMIN/df, chi-square to degrees of freedom ratio; CFI, Comparative Fit Index; TLI, Tucker–Lewis Index; RMSEA, Root Mean Square Error of Approximation; SRMR, Standardized Root Mean Square Residual.

#### 3.2.1 M1. Unidimensional model

This model specified all ten items loading onto a single latent perceived stress factor, consistent with the original conceptualization proposed by Cohen's (1988) and supported in previous studies (e.g., Luft et al., 2007; Mitchell et al., 2008; Santos-Vitti et al., 2024).

#### 3.2.2 M2. Correlated two-factor model

This model specified two correlated latent dimensions: Negative Stress (negatively worded items) and Coping (positively worded items). It was included because this structure emerged from the exploratory factor analysis conducted in the present study and has been consistently supported in previous psychometric studies of the PSS-10 (e.g., Dominguez-Lara et al., 2022).

#### 3.2.3 M3. Bifactor model

This model specified one general perceived stress factor (G) underlying all items, together with two orthogonal specific factors: Negative stress and Coping. The negatively worded items (Items 1, 2, 3, 6, 9, and 10) additionally loaded on the Negative stress factor, whereas the positively worded items (Items 4, 5, 7, and 8) loaded on the Coping factor, following the bifactor approach proposed by Denovan et al. (2017) and Juárez-García et al. (2023).

Among the evaluated models, the bifactor structure (M3) demonstrated the best relative fit to the data, with excellent incremental fit indices (CFI = 0.990, TLI = 0.982) and a low SRMR (0.043). However, the RMSEA remained above conventional cutoff criteria (RMSEA = 0.098, 90% CI [0.096, 0.101]). Therefore, the bifactor model was retained as the best-fitting factorial specification among the competing models evaluated because it consistently outperformed the alternative models across multiple fit indices and was further supported by the bifactor-specific indices presented below. Nevertheless, the elevated RMSEA indicates that the overall model fit remains imperfect and should be interpreted with caution, suggesting that the factorial structure of the PSS-10 may not be fully captured by any of the factorial models evaluated. In contrast, the unidimensional model (M1) exhibited clearly inadequate fit, whereas the correlated two-factor model (M2) showed acceptable comparative fit indices but poorer overall fit due to its elevated RMSEA and SRMR values.

**Figure 1:**
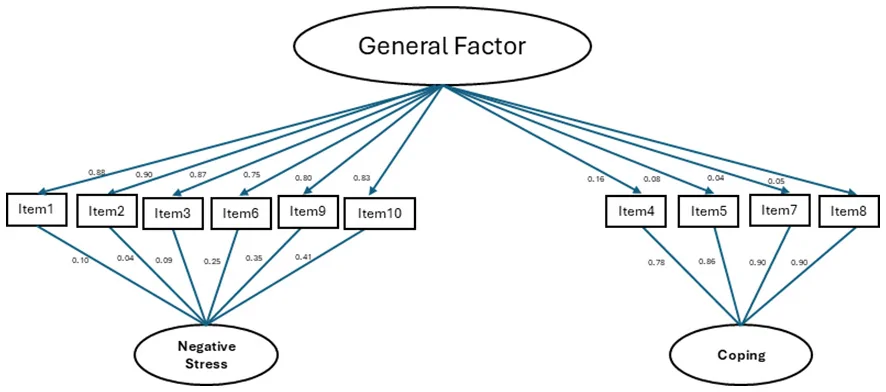
Standardized factor loadings of the bifactor model for the Perceived Stress Scale (PSS-10).

Standardized factor loadings for the retained bifactor model are presented in Figure 1. As illustrated in the diagram, most items loaded strongly on the General Factor, particularly the negatively worded items (Items 1, 2, 3, 6, 9, and 10), with standardized loadings generally above 0.70. These findings support the existence of a broad common latent dimension underlying perceived stress responses. In contrast, the positively worded items demonstrated comparatively weaker loadings on the General Factor but substantial loadings on the Coping specific factor (Item 4 = 0.778, Item 5 = 0.858, Item 7 = 0.896, Item 8 = 0.904), suggesting the presence of residual variance associated with item wording and perceived coping/self-efficacy content.

Bifactor-specific indices were subsequently examined to evaluate the relative contribution of the general and specific factors underlying the PSS-10 (see Table 4). The results supported the predominance of a strong general perceived stress factor. Specifically, the General Factor accounted for the largest proportion of common variance (ECV = 0.561), indicating that more than half of the common variance among the items was explained by a single overarching perceived stress dimension.

**Table 4:** Bifactor-specific indices for the PSS-10 (general factor and two specific factors).

Model	Factor/nested factors	ECV	ω	ωh	*H*	PUC
Hierarchical general factor and two nested factors	General	0.561	0.945	0.645	0.940	0.533
	Factor 1	0.049	0.950	0.054	0.297	
	Factor 2	0.319	0.922	0.914	0.928	

ECV (S&E), Explained Common Variance calculated using the formula proposed by Stucky and Edelen (2015); ω, omega coefficient; ωh, hierarchical omega coefficient; H, construct replicability index; PUC, Percentage of Uncontaminated Correlations.

By contrast, the Negative Stress specific factor accounted for only a small proportion of unique common variance (ECV = 0.049), indicating a limited contribution beyond the General Factor. In comparison, the Coping specific factor accounted for a substantially larger proportion of unique common variance (ECV = 0.319), suggesting that this dimension retained a meaningful amount of variance not captured by the General Factor.

The Percentage of Uncontaminated Correlations (PUC = 0.533) indicated a moderate proportion of correlations primarily attributable to the General Factor. Taken together, these findings indicate that the general perceived stress factor accounts for the largest proportion of common variance and supports the use of the total PSS-10 score as an overall indicator of perceived stress. At the same time, the substantial reliability and construct replicability of the Coping specific factor suggest that this dimension retains meaningful variance beyond the general factor, whereas the Negative Stress specific factor contributes relatively little unique reliable variance after controlling for the general factor. Accordingly, the bifactor indices support a nuanced interpretation in which the total score is informative, while the Coping dimension may also capture substantively relevant information.

### 3.3 Reliability

The reliability analyses demonstrated adequate-to-excellent internal consistency for the perceived stress scale and its latent dimensions (see Table 4). The total scale showed satisfactory reliability coefficients, with omega coefficient (ω = 0.945), supporting the consistency of the overall measure.

Within the bifactor structure, the general factor demonstrated excellent reliability (ω = 0.945), indicating that the total PSS-10 score provides a highly reliable estimate of perceived stress. Furthermore, the hierarchical omega coefficient for the general factor (ω*h* = 0.645) suggested that a substantial proportion of the reliable variance in total scores was attributable to the common latent dimension.

Regarding the specific factors, the Negative stress factor showed a very low hierarchical omega coefficient (ω*h* = 0.054), indicating limited reliable variance after controlling for the general factor. In contrast, the Coping factor retained a high hierarchical omega coefficient (ω*h* = 0.914), suggesting greater residual specificity within the bifactor solution.

Construct replicability indices also supported the stability of the general factor (*H* = 0.940) and the Coping factor (*H* = 0.928), whereas the Negative stress factor showed poor replicability (*H* = 0.297). Overall, these findings indicate that the PSS-10 demonstrates strong reliability primarily at the level of the general perceived stress factor.

### 3.4 Factorial invariance of the model across sex

The measurement invariance of the PSS-10 across sex was examined using multigroup confirmatory factor analysis (see Table 5). First, the baseline models estimated separately for men and women showed an acceptable overall pattern of fit, suggesting that the factorial structure of the instrument was reasonably replicated across groups. The configural invariance model (MC), which evaluates whether the same factorial structure is maintained across groups without equality constraints, showed favorable comparative and residual fit indices (CFI = 0.990, SRMR = 0.040), although its RMSEA (0.094) indicated that the absolute fit remained imperfect.

**Table 5:** Measurement invariance of the perceived stress scale across sex.

Model	χ^2^	df	C-M	*Δχ* ^2^	Δ*df*	CFI	ΔCFI	SRMR	RMSEA [CI 90%]	ΔRMSEA
Entire Group	1,931	25	–	–	–	0.990	–	0.042	0.098	**–**
Men	7,88.6	25	–	–	–	0.993	–	0.043	0.085	**–**
Women	1,066	25	–	–	–	0.984	–	0.036	0.105	**–**
MC	1,816.2	50	–	–	–	0.990	–	0.040	0.094	–
MM	3,735	67	MM–MC	1,918.8	17	0.978	0.012	0.064	0.117	0.023
SC	2,905.5	94	SC–MM	−829.5	27	0.983	0.005	0.060	0.086	−0.031

χ^2^, chi-square statistic; df, degrees of freedom; C–M, comparison between factorial invariance models; CFI, Comparative Fit Index; SRMR, Standardized Root Mean Square Residual; RMSEA, Root Mean Square Error of Approximation (90% confidence interval); Δ, change between nested models. Models: MC, Configural Model; MM, Metric Model; SC, Scalar Model.

The scalar model is reported for completeness but is not interpreted as evidence of scalar invariance because the metric model did not satisfy the prespecified change-in-fit criteria (ΔCFI ≤ 0.010; ΔRMSEA ≤ 0.015). Under WLSMV estimation, robust fit statistics may exhibit non-monotonic patterns across increasingly constrained models.

Subsequently, metric invariance (MM) was evaluated by constraining factor loadings to be equal across sex. Although the chi-square difference test was statistically significant (Δχ^2^ = 1,918.8, Δdf = 17), this result should be interpreted cautiously because chi-square statistics are highly sensitive to large sample sizes and robust WLSMV estimation. More importantly, the changes in fit indices exceeded the prespecified criteria (ΔCFI = 0.012; ΔRMSEA = 0.023), indicating that full metric invariance was not supported according to the decision criteria adopted in the present study.

A scalar model (SC), which additionally constrained item intercepts, was also estimated and is reported for completeness. Although this model yielded comparatively better fit indices than the metric model (CFI = 0.983, RMSEA = 0.086), robust fit statistics obtained with the WLSMV estimator may exhibit non-monotonic patterns across increasingly constrained models. Therefore, because full metric invariance was not established, the scalar model is not interpreted as evidence of scalar invariance.

Overall, the present findings provide limited evidence regarding measurement equivalence across sex. Consequently, although the factorial structure of the PSS-10 appears broadly comparable between men and women, comparisons of observed scores across sex should be interpreted cautiously until stronger evidence of measurement invariance is available.

### 3.5 Convergent validity with transdiagnostic variables

Pearson correlation analyses were conducted to examine the associations between perceived stress (total score and its two dimensions) and theoretically related transdiagnostic variables, including loneliness, psychological inflexibility, depressive symptoms, and impulsivity (see Table 6).

**Table 6:** Pearson correlations providing evidence of validity based on relationships with external variables.

Variable	Total stress	Negative stress	Coping
Loneliness (UCLA-3)	0.582^*^	0.677^*^	0.044^*^
Psychological Inflexibility (AAQ-II)	0.711^*^	0.756^*^	0.148^*^
Depressive Symptoms (PHQ-9)	0.698^*^	0.749^*^	0.136^*^
Total Impulsivity (Biss-11)	0.579^*^	0.502^*^	0.272^*^
Cognitive Impulsivity (Biss-11)	0.539^*^	0.460^*^	0.263^*^
Motor Impulsivity (Biss-11)	0.405^*^	0.489^*^	0.007
Non-planning Impulsivity (Biss-11)	0.413^*^	0.229^*^	0.366^*^

UCLA-3, UCLA Loneliness Scale–Short Version; AAQ-II, Acceptance and Action Questionnaire-II; PHQ-9, Patient Health Questionnaire-9; BIS-11, Barratt Impulsiveness Scale-11.

^*^p < 0.001.

The total perceived stress score showed positive and statistically significant correlations with all transdiagnostic variables, providing evidence of convergent validity. Correlation magnitudes ranged from moderate to large (Cohen's, 1988), with the strongest associations observed for psychological inflexibility (*r* = 0.711, *p* < 0.001) and depressive symptoms (*r* = 0.698, *p* < 0.001), followed by loneliness (*r* = 0.582, *p* < 0.001) and total impulsivity (*r* = 0.579, *p* < 0.001). Similar patterns were observed across the three impulsivity subdimensions.

The Negative Stress dimension demonstrated even stronger associations with these variables, with correlations ranging from 0.229 to 0.756 (all ps < 0.001). The largest coefficients were observed for psychological inflexibility (*r* = 0.756), depressive symptoms (*r* = 0.749), and loneliness (*r* = 0.677), supporting the interpretation of this dimension as reflecting the maladaptive component of perceived stress.

In contrast, the Coping dimension exhibited weak correlations with the transdiagnostic variables (*r* = 0.007 to 0.366). Although most associations reached statistical significance because of the large sample size, their magnitudes were small, and the association with motor impulsivity was not significant (*r* = 0.007, *p* = 0.392). This pattern suggests that the coping dimension captures variance that is relatively independent of psychological negative stress and related transdiagnostic processes.

Overall, these findings provide evidence of convergent validity for the PSS-10. As hypothesized, the total score and the Negative Stress dimension were strongly associated with key transdiagnostic processes and indicators of psychological maladjustment, whereas the Coping dimension demonstrated substantially weaker associations, supporting its conceptual distinctiveness.

### 3.6 Construct validity through contrasted groups

The contrasted-groups analysis revealed statistically significant sex differences across all items and dimensions of the perceived stress scale (all *p* < 0.001), providing additional evidence of construct validity (see Table 7). Specifically, female participants consistently reported higher scores than males on items associated with negative stress perception, particularly on Items 1, 2, 3, 6, 9, and 10. The largest differences were observed for Item 3 (η^2^ = 0.187), followed by Item 2 (η^2^ = 0.129), Item 1 (η^2^ = 0.117), Item 9 (η^2^ = 0.114), and Item 10 (η^2^ = 0.112), indicating moderate-to-large effect sizes. These findings are theoretically coherent with previous literature showing that women tend to report higher levels of perceived stress and emotional negative stress than men.

**Table 7:** Sex differences in perceived stress scale scores: evidence of construct validity through the contrasted-groups method.

Items	Total sample (*N* = 16,074) M (±SD)	Female (*n* = 7,528) *M* (±SD)	Male (*n* = 8,546) *M* (±SD)	*F*	*p*	*η^2^*
Item 1	1.218 (±1.134)	1.601 (±1.134)	0.881 (±1.021)	1,061.176	< 0.001	0.117
Item 2	1.101 (±1.187)	1.450 (±1.219)	0.794 (±1.069)	1,191.017	< 0.001	0.129
Item 3	1.755 (±1.311)	2.285 (±1.188)	1.289 (±1.235)	1,850.492	< 0.001	0.187
Item 4	1.752 (±1.399)	1.645 (±1.224)	1.846 (±1.531)	1,44.736	< 0.001	0.018
Item 5	1.650 (±1.283)	1.632 (±1.130)	1.666 (±1.403)	255.782	< 0.001	0.031
Item 6	1.336 (±1.207)	1.685 (±1.164)	1.029 (±1.160)	831.953	< 0.001	0.094
Item 7	1.677 (±1.308)	1.613 (±1.130)	1.733 (±1.444)	211.252	< 0.001	0.026
Item 8	1.669 (±1.295)	1.657 (±1.139)	1.679 (±1.417)	238.986	< 0.001	0.029
Item 9	1.419 (±1.204)	1.796 (±1.178)	1.086 (±1.127)	1,032.069	< 0.001	0.114
Item 10	1.217 (±1.210)	1.578 (±1.236)	0.900 (±1.092)	1,017.398	< 0.001	0.112
Negative stress	8.047 (±6.106)	10.395 (5.845)	5.978 (±5.560)	1,700.123	< 0.001	0.175
Coping	6.748 (±4.592)	6.548 (±3.909)	6.924 (±5.560)	267.037	< 0.001	0.032
Total	14.794 (±7.448)	16.943 (±7.293)	12.902(±7.060)	1,438.814	< 0.001	0.152

N, sample size; M, mean; SD, standard deviation; F, ANOVA F statistic; p, significance level; η^2^, eta squared effect size.

At the dimensional level, women obtained significantly higher scores on the Negative Stress factor (*M* = 10.395, SD = 5.845) compared to men (*M* = 5.978, SD = 5.560), with a large effect size (*F* = 1,700.123, η^2^ = 0.175). Likewise, the total perceived stress score was significantly higher among women (*M* = 16.943, SD = 7.293) than among men (*M* = 12.902, SD = 7.060), also demonstrating a substantial effect size (η^2^ = 0.152). In contrast, men showed slightly higher scores on the Coping dimension (*M* = 6.924, SD = 5.560) relative to women (*M* = 6.548, SD = 3.909), although the magnitude of this difference was small (η^2^ = 0.032).

Overall, the consistency and direction of these differences support the discriminative capacity of the scale and provide evidence of construct validity through the contrasted-groups method. The instrument successfully differentiated between groups that, according to prior theoretical and empirical evidence, are expected to differ in perceived stress levels and coping experiences.

### 3.7 Descriptive analysis of the best-fitting bifactor model of the PSS-10

Descriptive analyses were conducted for the total perceived stress score and the dimensions derived from the retained bifactor model. The total perceived stress score presented a prorated mean of 1.479 (SD = 0.745), whereas the Negative Stress dimension showed a mean score of 1.341 (SD = 1.018). The Coping dimension obtained the highest mean score (*M* = 1.687, SD = 1.148), suggesting that participants generally reported greater perceived coping/self-efficacy resources relative to negative stress experiences.

The distributions of the three variables showed adequate variability, with observed scores ranging from 0 to 4 across all dimensions. Median values were close to their corresponding means, indicating broadly symmetric distributions. Specifically, the median scores were 1.600 for Total Perceived Stress, 1.167 for Negative Stress, and 1.500 for Coping. The interquartile ranges indicated moderate score dispersion across the three variables, with the Negative Stress dimension exhibiting the widest interquartile range, whereas the Coping dimension showed the greatest overall variability as reflected by its standard deviation.

**Figure 2:**
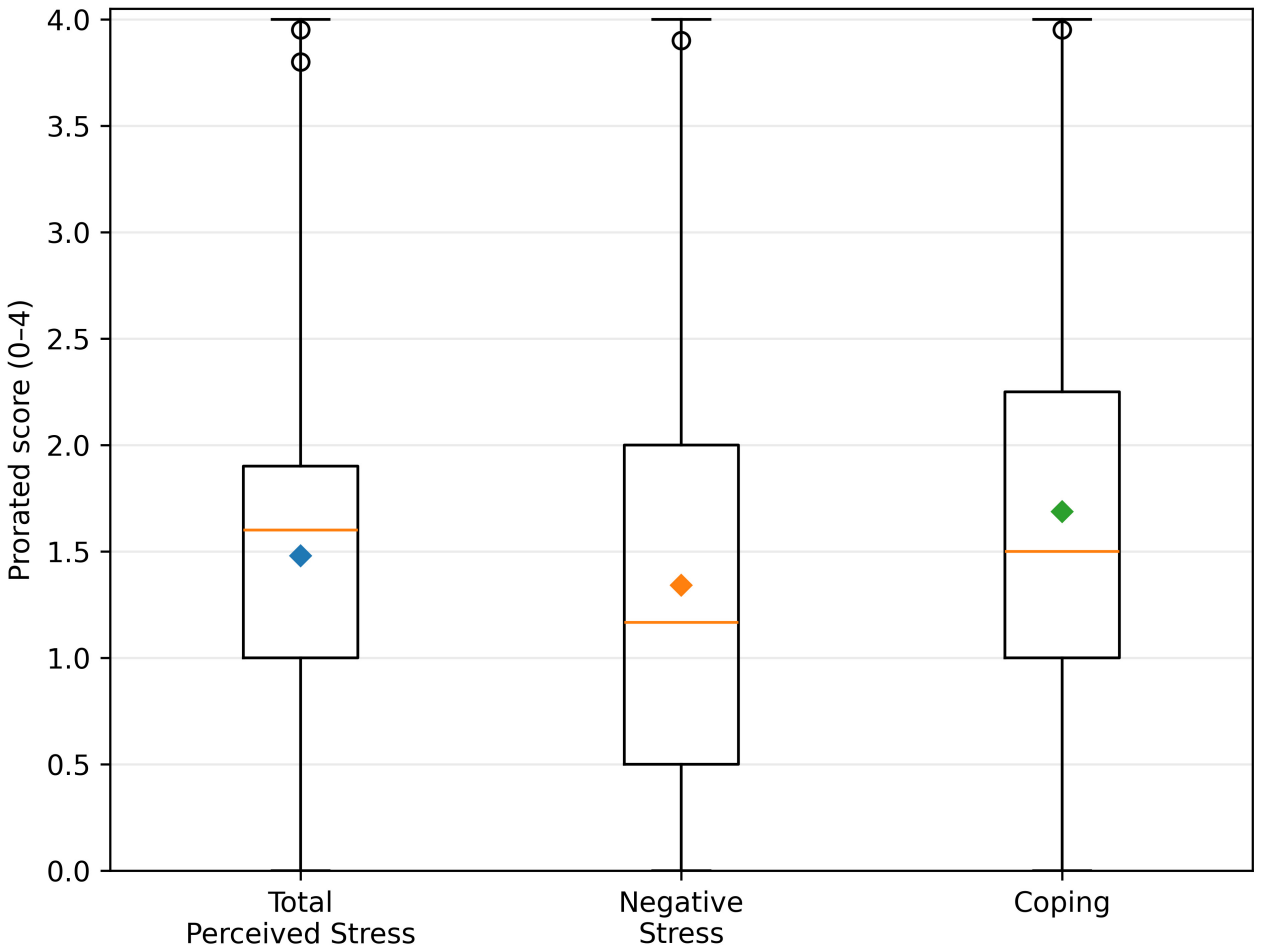
Distribution of prorated scores for the retain bifactor model.

Skewness values ranged from 0.166 to 0.531, indicating only slight positive asymmetry, whereas kurtosis values ranged from −0.553 to −0.101, suggesting mildly platykurtic distributions. Overall, these findings indicate that the score distributions did not exhibit substantial deviations from normality. The boxplots (Figure 2) were consistent with these descriptive statistics, showing relatively homogeneous score distributions across the three variables and only slight positive asymmetry. Although several upper-range outlying observations were identified, they were not extreme and are commonly expected in large population-based samples. No substantial floor or ceiling effects were observed, indicating that the response categories adequately captured variability in perceived stress and coping levels.

Taken together, these descriptive findings support the suitability of the data for subsequent confirmatory factor analyses and provide additional evidence for the psychometric adequacy of the retained bifactor structure of the PSS-10 in this Ecuadorian adult sample.

## 4 Discussion

The present study aimed to examine the psychometric properties of the Perceived Stress Scale (PSS-10) in a large sample of Ecuadorian adults. The results provide robust evidence for its factorial validity, reliability, and convergent validity with theoretically related transdiagnostic constructs, while providing only limited evidence regarding measurement invariance across sex.

First, the exploratory factor analysis supported a two-dimensional structure comprising the Negative Stress and Coping dimensions, a finding consistent with previous studies conducted across diverse cultural contexts (Dominguez-Lara et al., 2022; Jorquera-Gutiérrez and Guerra-Díaz, 2023; Makhubela, 2022). However, the confirmatory factor analyses indicated that the bifactor model provided the best fit to the data, outperforming both the unidimensional and correlated two-factor models.

Although the bifactor model yielded an RMSEA value (0.098; 90% CI [0.096, 0.101]) exceeding conventional cutoff criteria, this finding should be interpreted within the context of the overall pattern of model fit rather than in isolation. Recent psychometric research has shown that RMSEA may overestimate model misfit in confirmatory factor analyses estimated using the WLSMV estimator with ordinal indicators, particularly when models include relatively few indicators, low degrees of freedom, or very large sample sizes (Shi et al., 2019; Xia and Yang, 2019). Under these conditions, RMSEA may indicate poorer absolute fit even when other fit indices support an acceptable model. Accordingly, current methodological recommendations recommend evaluating model fit using multiple complementary indices rather than relying exclusively on RMSEA (Shi et al., 2019; Xia and Yang, 2019).

Therefore, although the elevated RMSEA should be acknowledged as a limitation of the present model, the convergence of the remaining fit indices (CFI = 0.990, TLI = 0.982, and SRMR = 0.043), together with the bifactor-specific indices, supports the bifactor solution as the best-fitting representation among the factorial models evaluated in the present sample. At the same time, the elevated RMSEA indicates that the overall model fit remains imperfect. Although the standardized factor loadings and bifactor-specific indices consistently supported the retained structure, the RMSEA suggests that some degree of localized model misfit may remain, indicating that the factorial structure of the PSS-10 may not be fully captured by the models evaluated. This interpretation is nevertheless consistent with previous psychometric studies indicating that the PSS-10 is characterized by a strong general perceived stress factor alongside specific factors primarily associated with positively and negatively worded items (Denovan et al., 2017; Perera et al., 2017; Reis et al., 2019; Juárez-García et al., 2023; Yilmaz Kogar and Kogar, 2024). Future research could examine alternative theoretically informed specifications, including correlated residuals among similarly worded items or exploratory structural equation modeling (ESEM), to determine whether these approaches provide a more comprehensive representation of the latent structure of perceived stress.

Accordingly, the present findings do not allow a definitive distinction between variance attributable to wording effects and variance reflecting substantive psychological processes. Both interpretations remain plausible, particularly for the Coping factor, and future studies should explicitly compare these competing explanations using alternative modeling approaches.

The bifactor-specific indices further supported this interpretation. The explained common variance (ECV = 0.561), together with the high hierarchical omega coefficient (ω*H* = 0.645) and construct replicability index (*H* = 0.940), indicates that a substantial proportion of the common variance among the items is accounted for by a single general factor of perceived stress (Chen et al., 2024; Guimarães Freitas et al., 2025; Jatic et al., 2023; Xiao et al., 2023).

However, the bifactor coefficients also indicate that the two specific factors do not contribute equally after accounting for the general factor. Whereas, the Negative Stress factor retained little unique reliable variance, the Coping factor remained highly reliable and replicable (ω*H* = 0.914; *H* = 0.928), suggesting that it captures substantively meaningful individual differences beyond the general perceived stress factor. Consequently, the present findings support the use of the total PSS-10 score as an overall indicator of perceived stress while also indicating that the Coping dimension may warrant consideration in studies specifically examining perceived control, coping resources, or related psychological processes (Lazarus and Folkman, 1984; Denovan et al., 2017; Juárez-García et al., 2023).

From a psychometric perspective, these findings suggest that the total PSS-10 score provides a useful overall indicator of perceived stress, consistent with previous bifactor studies reporting a predominant general perceived stress factor (Chen et al., 2024; Guimarães Freitas et al., 2025; Jatic et al., 2023; Xiao et al., 2023). At the same time, the strong reliability and construct replicability of the Coping factor indicate that this dimension may reflect substantively meaningful variance in addition to any variance associated with positively worded items, a finding that is also compatible with previous psychometric studies highlighting the persistence of specific variance in positively worded PSS-10 items (Denovan et al., 2017; Juárez-García et al., 2023; Yilmaz Kogar and Kogar, 2024). Conversely, the Negative Stress specific factor demonstrated little reliable variance after accounting for the general factor, providing limited support for its interpretation as an independent subscale. Overall, these findings favor a nuanced interpretation of the PSS-10 in which the general factor predominates, while the Coping dimension may also convey meaningful information depending on the objectives of the assessment.

The reliability analyses provided further support for the psychometric adequacy of the PSS-10. The total score demonstrated excellent reliability (ω = 0.945), indicating that the instrument yields highly consistent scores. However, within the bifactor framework, the hierarchical omega coefficient (ω*H* = 0.645) indicates that approximately two-thirds of the reliable variance in the total score is attributable to the general perceived stress factor, whereas the remaining reliable variance is associated with the specific factors. Thus, although the total score provides a reliable overall indicator of perceived stress, its interpretation should acknowledge the contribution of residual specific variance, particularly that associated with the Coping dimension. Likewise, the general perceived stress factor demonstrated excellent construct replicability (*H* = 0.940), supporting the stability of the general construct across items. Consistent with this interpretation, the Coping specific factor exhibited high reliability (ω = 0.922; ω*H* = 0.914) and construct replicability (*H* = 0.928), suggesting that it captures reliable variance beyond the general perceived stress factor. In contrast, the Negative Stress specific factor retained little unique reliable variance (ω*H* = 0.054; *H* = 0.297), providing limited support for its interpretation as an independent subscale.

These findings suggest that, although the general perceived stress factor accounts for the largest proportion of reliable variance, the Coping dimension also captures meaningful residual variance that may contribute additional information in studies focusing on perceived coping resources or perceived control. This interpretation is consistent with previous bifactor studies reporting a predominant general perceived stress factor while also recognizing that specific dimensions may retain meaningful variance (Denovan et al., 2017; Juárez-García et al., 2023; Reis et al., 2019). Accordingly, the present findings support the use of the total PSS-10 score as an overall indicator of perceived stress, while acknowledging that it reflects both the general factor and residual reliable variance associated with the specific factors, particularly the Coping dimension (Denovan and Dagnall, 2025; Ferreira et al., 2025; Yilmaz Kogar and Kogar, 2024).

The evidence regarding measurement invariance across sex should be interpreted cautiously. Although the configural model showed favorable comparative and residual fit indices, its elevated RMSEA indicated imperfect absolute fit. More importantly, the transition from the configural to the metric model exceeded the prespecified change-in-fit criteria, indicating that full metric invariance was not supported in the present sample. Although the scalar model yielded comparatively better fit indices than the metric model, robust WLSMV estimation may produce non-monotonic changes in fit statistics across increasingly constrained models. Therefore, the scalar model was reported for completeness but was not interpreted as evidence of scalar invariance. Overall, the present findings provide limited evidence regarding the comparability of the PSS-10 across sex.

These results are only partially consistent with previous studies that have reported measurement invariance of the PSS-10 across sex in different populations (Boluarte-Carbajal et al., 2023; Chen et al., 2024; Denovan and Dagnall, 2025; Juárez-García et al., 2023; Santos-Vitti et al., 2024; Yilmaz Kogar and Kogar, 2024). For example, Chen et al. (2024) reported latent mean invariance across gender among adolescents, whereas Boluarte-Carbajal et al. (2023) found evidence of metric invariance in a Peruvian sample. The discrepancies across studies may reflect differences in population characteristics, cultural context, sample composition, or analytical strategies. Consequently, further research is needed to clarify the measurement equivalence of the PSS-10 across diverse populations, including the evaluation of partial invariance and alternative approaches for ordered-categorical indicators.

Regarding known-groups validity, women reported significantly higher levels of perceived stress and Negative Stress than men, whereas men obtained slightly higher scores on the Coping dimension. These findings are consistent with the international literature, which has consistently documented higher levels of perceived stress, anxiety, and psychological negative stress among women (Bamert et al., 2025; Chen et al., 2024; Graves et al., 2021; Maqsood et al., 2024; Matud, 2004; Nolen-Hoeksema, 2012). According to the transactional model of stress proposed by Lazarus and Folkman (1984), these sex differences may reflect variations in the cognitive appraisal of stressors, the availability and use of coping resources, and differential exposure to social and emotional demands (Graves et al., 2021; Maqsood et al., 2024). However, because full metric invariance across sex was not established in the present study, these findings should be interpreted as descriptive differences in observed scores rather than definitive evidence of latent differences between men and women.

The correlational analyses provided robust evidence of convergent validity. As hypothesized, perceived stress was positively associated with loneliness, psychological inflexibility, depressive symptoms, and impulsivity, with correlations ranging from moderate to strong. Particularly noteworthy were the strong associations with psychological inflexibility (*r* = 0.711) and depressive symptoms (*r* = 0.698), supporting the close relationship between perceived stress and key psychological processes underlying psychological negative stress.

These findings are consistent with previous studies conducted in Ecuador and other countries, which have identified perceived stress as a construct closely associated with multiple indicators of mental health (Reis et al., 2019; Ruisoto et al., 2020; Vaca-Gallegos et al., 2025; Yao et al., 2023; Zeas-Sigüenza et al., 2023). In particular, the strong association observed with psychological inflexibility is especially relevant from the perspective of Acceptance and Commitment Therapy (ACT), which conceptualizes experiential avoidance and psychological inflexibility as transdiagnostic vulnerability processes underlying a wide range of emotional disorders (Hayes et al., 2006). Individuals experiencing higher levels of perceived stress may be more likely to engage in experiential avoidance and rigid behavioral patterns, thereby maintaining or exacerbating cycles of psychological negative stress.

Similarly, the association between perceived stress and depressive symptoms is consistent with substantial evidence identifying perceived stress as a major risk factor for the onset and maintenance of depression. Likewise, the relationship with loneliness suggests that individuals who perceive their lives as unpredictable, uncontrollable, or overwhelming also experience greater interpersonal difficulties and lower levels of social connectedness. Together, these findings underscore the close relationship between perceived stress and both psychological and social wellbeing.

Another noteworthy finding was the association between perceived stress and impulsivity. Although this relationship has received comparatively little attention in psychometric studies of the PSS-10, the present results suggest that elevated levels of perceived stress may compromise self-regulatory processes and behavioral control (Maqsood et al., 2024). From a neuropsychological perspective, prolonged exposure to stress has been shown to disrupt executive functioning, thereby increasing the likelihood of impulsive responses and less deliberative decision-making (Yao et al., 2023).

Taken together, these findings support the conceptualization of perceived stress as a transdiagnostic factor. The strength and consistency of its associations with diverse constructs (including depression, psychological inflexibility, loneliness, and impulsivity) suggest that perceived stress occupies a central position within the psychological processes underlying multiple forms of emotional negative stress. This interpretation is consistent with recent theoretical and empirical evidence proposing perceived stress as a common mechanism involved in a broad range of mental health problems (Ruisoto et al., 2022; Vaca-Gallegos et al., 2025; Yao et al., 2023).

Several limitations of this study should be acknowledged. First, the use of a non-probability sample limits the generalizability of the findings to the broader Ecuadorian population. Although participants were recruited from all four geographical regions of Ecuador, most respondents came from the Andean and Coastal regions, reflecting the country's population distribution and the greater accessibility of these areas during data collection. While this regional imbalance may limit the generalizability of the findings to the Amazon and Galápagos regions, the primary objective of this study was to evaluate the psychometric properties of the PSS-10 rather than to compare perceived stress across geographical regions. Consequently, this distribution is less likely to have materially affected the estimation of the instrument's factorial structure and reliability. Second, the cross-sectional design precludes causal inferences regarding the relationships among the study variables. Finally, all measures were based on self-report, which may have increased the influence of social desirability bias and common method variance (Podsakoff et al., 2003; Van de Mortel, 2008; Latkin et al., 2017). Future research should examine the measurement invariance and psychometric performance of the PSS-10 across Ecuador's geographical regions using more regionally balanced samples, assess the temporal stability of the bifactor structure (Denovan and Dagnall, 2025), evaluate the predictive validity of the PSS-10 using longitudinal designs, and further investigate the role of perceived stress within broader transdiagnostic models incorporating variables such as anxiety, resilience, psychological flexibility, and wellbeing (Yilmaz Kogar and Kogar, 2024).

Future studies should examine whether alternative specifications, such as models incorporating theoretically justified correlated residuals among similarly worded items or exploratory structural equation modeling (ESEM), provide a more accurate representation of the latent structure of the PSS-10.

Despite these limitations, the present study represents one of the largest psychometric evaluations of the PSS-10 conducted in Latin America. The findings provide robust evidence that the scale is best represented by a bifactor structure characterized by a dominant general factor, adequate reliability, and meaningful associations with key mental health indicators, although measurement invariance across sex was only partially supported. Accordingly, the PSS-10 can be recommended as a valid and reliable instrument for research, clinical practice, epidemiological studies, and mental health surveillance initiatives in Ecuador, with sex-based comparisons interpreted cautiously pending further invariance evidence.

### 4.1 Practical implications

The present findings have several practical implications for research, clinical practice, and public mental health in Ecuador. First, the availability of a psychometrically robust version of the PSS-10 provides researchers and mental health professionals with a reliable and culturally appropriate instrument for assessing perceived stress in the Ecuadorian adult population. The availability of culturally validated instruments is essential for ensuring accurate measurement and meaningful interpretation of psychological constructs across different populations (American Educational Research Association, 2014). Accurate assessment is particularly important because perceived stress has consistently been identified as one of the central transdiagnostic processes underlying psychological maladjustment and has been associated with a broad range of adverse mental health outcomes (Cohen et al., 1983; Lee, 2012; Ruisoto et al., 2022).

Evidence from Ecuador indicates that perceived stress plays a central role in psychological functioning. Previous studies have shown that it is one of the strongest predictors of depressive symptoms and constitutes a key mechanism linking loneliness and psychological inflexibility with depression within transdiagnostic models of mental health (Ruisoto et al., 2022). Likewise, recent evidence suggests that perceived stress mediates the relationship between positive psychological resources and symptoms of anxiety and depression, highlighting its relevance as a common psychological process underlying emotional distress (Quinde et al., 2026).

Consequently, the availability of a valid and reliable measure of perceived stress may facilitate the early identification of individuals at greater risk of psychological distress and support the implementation, monitoring, and evaluation of prevention and mental health promotion programs in educational, healthcare, and community settings (Lee, 2012). Although the present findings provide limited evidence regarding the comparability of the PSS-10 across sex, additional research is needed to further clarify measurement equivalence before drawing definitive conclusions about sex-based comparisons. Nevertheless, the availability of normative psychometric evidence from a large community sample provides a solid foundation for future longitudinal, cross-cultural, and intervention studies aimed at refining the measurement of perceived stress and advancing the understanding of its role in mental health.

## 5 Conclusions

The present findings provide robust evidence supporting the reliability and validity of the Ecuadorian version of the Perceived Stress Scale (PSS-10) in a large community sample of adults. Among the alternative factorial structures evaluated, the bifactor model provided the best overall representation of the data and was further supported by bifactor-specific indices. Taken together, the findings support the use of the total PSS-10 score as an overall indicator of perceived stress. At the same time, the substantial reliability and construct replicability of the Coping specific factor indicate that this dimension captures meaningful variance beyond the general factor and should not be interpreted solely as a negligible wording effect, whereas the Negative Stress factor showed limited unique reliability after accounting for general perceived stress. However, the elevated RMSEA indicates that the overall model fit remains imperfect and suggests that the latent structure of the instrument may not be fully captured by the factorial models evaluated. Furthermore, the measurement invariance analyses provided limited evidence regarding measurement equivalence across sex because full metric invariance was not established according to the prespecified criteria. Accordingly, sex-based score comparisons should be interpreted cautiously until additional evidence of measurement equivalence becomes available. Therefore, the present findings should be interpreted considering the overall pattern of psychometric evidence rather than any single model fit index.

The observed associations between perceived stress and loneliness, psychological inflexibility, depressive symptoms, and impulsivity are consistent with previous research and provide additional evidence of validity based on relationships with theoretically relevant constructs. Overall, the findings suggest that the PSS-10 is a useful instrument for assessing perceived stress in Ecuadorian adults, although additional psychometric research is warranted to further clarify its latent structure and measurement equivalence across groups. Future studies should continue evaluating alternative theoretically informed specifications, including models with correlated residuals among similarly worded items, exploratory structural equation modeling (ESEM), and further analyses of measurement equivalence across diverse populations to refine the assessment of perceived stress and advance the understanding of its dimensional structure.

## Data Availability

The raw data supporting the conclusions of this article will be made available by the authors, without undue reservation.
